# The rationale for cancer to be made a notifiable disease in India

**DOI:** 10.5588/pha.25.0006

**Published:** 2025-06-04

**Authors:** R. Singh, A.L. Frank

**Affiliations:** Department of Environmental and Occupational Health, Dornsife School of Public Health, Drexel University, Philadelphia, PA, USA.

**Keywords:** risk factors, National Cancer Registry, mesothelioma, ICMR-NCDIR

## Abstract

Cancer cases are rising in India. However, despite attempts to make cancer a notifiable disease, the Ministry of Health in India has resisted this. Their reasoning is that cancer is not a communicable disease and does not have community spread. We highlight flaws in the logic of this argument and highlight how a legal mandate to report cases would give impetus to the decades-old National Cancer Registry Program. Robust record keeping would allow real-time epidemiological analysis and highlight those areas where priority could be given to both prevent and treat cancer.

Close to half of deaths due to cancer can be avoided by prevention and control of risk factors.^1^ These risk factors include smoking, air pollution and mineral dust (such as asbestos). To manage these risks, a government must first have data on cancer incidence, prevalence, morbidity and mortality rates. This requires proper record-keeping in a national registry, which would allow for the appropriate allocation of resources to those areas where priority should be given to reduce cancer rates. A registry would also allow the data to be shared with national and international agencies and the development sector, to better focus on the priority areas. This data is key to providing real-time epidemiological evidence and establishing whether there is a causal link between risk factors and the disease.^2^ Cancer record-keeping is made possible through registries, operated either individually by hospitals or as a collective of many hospitals (where a large hospital records cases from other smaller hospitals in its catchment area in a shared database). Since 1981, the Government of India’s National Cancer Registry Program is run by the Indian Council of Medical Research’s National Centre for Disease Informatics and Research, Bengaluru (ICMR-NCDIR). However, the ICMR-NCDIR registry covers only 16% of the country’s population. This was noted by a parliamentary committee on health set up to deal with cancer, which expressed ‘it’s deep displeasure’ with the current state of affairs.^3^ This limited coverage by the National Cancer Registry Program is likely due to the absence of a legal mandate for hospitals and healthcare providers to provide this data. The solution, therefore, is to make cancer a notifiable disease. Once enshrined in law, medical practitioners would be required to report cases to government authorities. This was also recommended by the ICMR-NCDIR in a 2020 policy brief, which states: *‘*Making cancer a notifiable disease to enable increased coverage by registries and establishment of registries in areas hitherto uncovered regions.’^4^

In 2024, in a case filed before the National Human Rights Commission of India (NHRC: 59/30/3/2024) on the matter of making cancer notifiable in India, the NHRC directed the Secretary of the Ministry of Health and Family Welfare (MoHFW) to ensure that such an action, as deemed appropriate, in the matter, is taken. The MoHFW replied to the NHRC as follows:

‘A notifiable disease is any disease that is required by law to be reported to government authorities. The collation of information allows the authorities to monitor the disease and provides early warning of possible outbreaks.

The World Health Organisation’s International Health Regulations require disease reporting to the WHO in order to help with its global surveillance and advisory role. Making a disease legally notifiable by doctors and health professionals allows intervention to control the spread of highly infectious diseases.

Cancer is a type of non-communicable disease. It is not an infectious disease. It does not spread from one person to another or also does not have any community spread. In present circumstances, it may not be declared as notifiable disease.’

Because of this response by the MoHFW, the parliamentary committee took back its recommendations.^5^ However, despite the position adopted by the MoHFW, many states in India recognise the importance of recording cancer data and 17 states have already made cancer notifiable at the state level. In the absence of public health legislation, some states took exceptional measures, which include using administrative orders to make cancer notifiable. Some states, such as Tamil Nadu, operate a comprehensive state-level registry programme.

The response by the MoHFW raises another important issue regarding communicable versus non-communicable diseases, and whether only communicable diseases should be notifiable. First, it should be noted that some cancers are known to be communicable diseases, for example cervical cancer is caused by transmission of human papillomavirus through skin-to-skin contact.^6^ Second, there is a major inconsistency in that the MoHFW directed a known non-communicable disease (i.e., snakebite) to be made a notifiable disease.^7^ The Tata Memorial Centre in Mumbai has an innovative semantic solution to this dilemma and recommended to the parliamentary committee that cancer be declared a ‘documentable disease’. In this way, it would still be compulsory for it to be reported, and rigorous record-keeping can still take place, even if the condition is non-communicable.^3^

A direction by the MoHFW to the states in India to make cancer notifiable (or the MoHFW itself to make cancer notifiable) is all the more relevant when India is facing the double burden of communicable as well as non-communicable diseases. Communities may be exposed to common factors that lead to many cancer cases, which should be monitored, recorded and acted upon. Cancer cases are on the rise in India (Figure). A recent report now calls India the ‘cancer capital of the world’ and highlights the ‘dire need to ramp up cancer screening’.^8^ Even this dramatic rise in cases may be incomplete because the available data likely represents only a small percentage of actual cases. This is illustrated by a study in which hospitals kept records of cancer cases and 75% of these hospitals were not part of the population-based cancer registries run by ICMR-NCDIR.^9^ Data collected by ICMR-NCDIR is reported to international agencies like the International Agency for Research on Cancer (IARC), which influences policy decisions, and should be comprehensive.^10,11^

**FIGURE.**
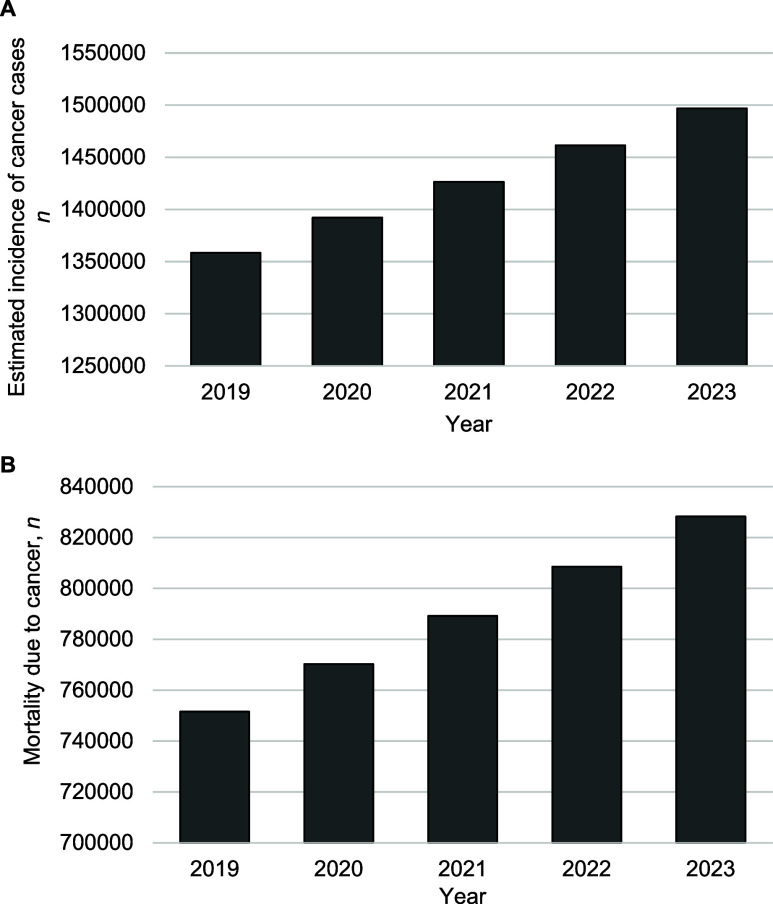
**A**) Estimated incidence and **B)** mortality for cancer cases in India 2019–2023 (ICD10:C00-C97). **Rajya Sabha Unstarred Question Number 907, Parliament of India; https://sansad.in/getFile/annex/265/AU907_33qphJ.pdf?source=pqars. ICD = International Classification of Diseases.

It is possible that the process of implementing a robust and accurate cancer registry would lead to a large number of cases being recorded, which might temporarily overwhelm government agencies. However, having such a registry would pave the way for effective cancer prevention (e.g., focussed screening) and treatment. This has been accomplished in Nordic countries (such as Denmark), where registries have been operating since 1942.^12-14^ In 1964, around the time when India’s cancer registry program’s predecessor started, a study reported that ‘there were several well-established cancer registries in Europe (Finland, Scotland, and Denmark), North America, and South America (Brazil), Asia (China and Singapore), and Oceania (Australia and NewZealand)’.^14^ In India, there has been an alarming lack of progress since then.

Notification of cancer at the national level would provide the impetus needed to finally make the decades-old Indian cancer registry programme work effectively to help reduce the impact of this dreadful disease.
